# The Attenuating Effect of Curcumin on Morphine Dependence in Rats: The Involvement of Spinal Microglial Cells and Inflammatory Cytokines

**DOI:** 10.22037/ijpr.2019.111701.13309

**Published:** 2019

**Authors:** Mohammad Abbas Sheikholeslami, Siavash Parvardeh, Shiva Ghafghazi, Taraneh Moini Zanjani, Masoumeh Sabetkasaei

**Affiliations:** *Department of Pharmacology, School of Medicine, Shahid Beheshti University of Medical Sciences, Tehran, Iran.*

**Keywords:** Morphine dependence, Withdrawal syndrome, Curcumin, Spinal cord, Microglial cells, Inflammatory cytokines, Rat

## Abstract

New evidence suggests an important role for spinal glial cells in the development of opioid dependence. Curcumin, a component of the Curcuma Longa, has shown to act as a suppressor of microglial cells. The main goal of this study was to explore the attenuating effects of curcumin on morphine dependence with a focus on spinal microglial cells and inflammatory cytokines. In order to induce morphine dependence in male Wistar rats, morphine was administered intraperitoneally (i.p.) once daily for 9 days in an increasing dose of 10, 20, and 40 mg/kg. Curcumin (2.5, 5, and 10 mg/kg, i.p.) was given from the days 10th to 18th. Naloxone-precipitated abstinence syndrome was used to assess the behavioral symptoms of morphine dependence. Immunofluorescence staining of Iba1 and ELISA test were used to measure spinal microglial activity and inflammatory cytokines levels, respectively.

The results showed that curcumin (2.5, 5, and 10 mg/kg) significantly decreased jumping, leaning, and diarrhea in morphine-dependent rats. In addition, the spinal concentration of TNF-α and IL-6 was reduced by curcumin (2.5, 5, and 10 mg/kg) significantly. Moreover, curcumin showed a potent attenuating effect on the number of Iba1 positive cells in rats which were subjected to morphine dependence. The results of this study demonstrated that curcumin exerts a remarkable reducing effect on morphine dependence in rats. The findings showed that the therapeutic effect of curcumin on morphine dependence is mediated through the suppression of activated microglial cells and reduction of inflammatory cytokines levels in the spinal cord.

## Introduction

Opioid analgesic drugs are the first-line pharmacologic agents for the treatment of severe pain. Continuous and prolonged administration of opioids often leads to physical dependence and tolerance which complicate pain management in patients (1). There is a serious concern that opioid physical dependence results in drug abuse and addiction (2). Furthermore, interruptions in opioid consumption elicit withdrawal syndrome which is considered a life-threatening situation (3). Besides, opioid dependence imposes a psychosocial burden and result in a significant decline in patients’ lifestyle indices (4). Accordingly, there has been plenty of attempts to overcome opioid dependence and addiction by focusing on the underlying mechanisms.

The hallmark antinociception and adverse reactions particularly physical dependence are predominantly mediated through the activation of μ opioid receptors (5). Several mechanisms have been explored for adaptive changes of μ opioid receptors following the long-term consumption of μ opioid receptor agonists. For instance, a significant increase in adenylyl cyclase activity along with a remarkable rise in cAMP levels and cAMP-dependent protein kinase as well as cAMP response element-binding protein (CREB) has been reported (6). 

Although μ opioid receptors are extensively distributed in the different areas of the brain (7), they are located on pre- and postsynaptic areas on nociceptive neurons in the dorsal spinal cord too. In addition, spinal glial cells including astrocytes and microglia have a remarkable number of μ opioid receptors (8). Recent evidence suggests that the spinal μ opioid receptors located in microglial cells are highly implicated in the induction and promotion of opioid dependence (6,9,10). In this regard, it has been shown that chronic administration of morphine causes microglial activation in rats’ spinal cord (9, 11, 12). The overactivity of microglial cells results in the production and secretion of large amounts of various products such as inflammatory cytokines, NO, and excitatory amino acids. Now it has been demonstrated that each of these mediators underlies the development of morphine dependence and the manifestation of morphine withdrawal syndrome (12). The current evidence shows that long-term exposure to morphine has a very close correlation to the overproduction of inflammatory cytokines by microglial cells (12, 13). For instance, chronic administration of morphine induces IL-1β expression which promotes physical dependence to morphine (14). Similar reports have been documented with respect to IL-6 pursuant to central or peripheral administration of opioids (12). Moreover, the long-term stimulation of μ opioid receptors in spinal glial cells contributes to the induction of physical dependence through the spinal matrix metalloproteinase-9 which is linked to CREB activation in the spinal cord (6).

Considering the role of spinal microglial cells in eliciting opioid dependence, it can be hypothesized that the suppressing the glial cells can attenuate physical dependence to opioids. This approach can efficiently interrupt the signaling pathways which ultimately cause opioid physical dependence. 

The application of medicinal plants in the management of opioid dependence has been taken into consideration over the last decade. An important part of studies has focused on the efficacy of major constituents of medicinal plants in the control of opioid dependence (15, 16). Among medicinal plants, Turmeric Hindi (Curcuma longa Linn), from the Zingiberaceae family, is emerging as an outstanding herb with unique pharmacological effects. Many therapeutic properties of turmeric have been attributed to its powdered root which is rich in curcumin. Curcumin [1,7-bis-(4-hydroxy-3-methoxyphenyl)-1,6-heptadiene-3,5-dione] is a diphenolic chemical which possesses therapeutic effect in various disorders such as cardiovascular, inflammatory, metabolic, and neurodegenerative diseases (17). There is evidence showing that curcumin dose-dependently reduces symptoms of withdrawal syndrome (18). Additionally, it has been found that curcumin attenuates morphine dependence through its antioxidant and anti-apoptotic effects in the hippocampus (19). Besides, it has been shown that curcumin prevents opioid tolerance and dependence by suppressing CaMKIIα activity (20). Many studies have demonstrated that curcumin has remarkable anti-inflammatory effects and suppresses the production and secretion of inflammatory cytokines potently (17, 21). For instance, current evidence indicates that the analgesic effect of curcumin in animal models of diabetic neuropathic pain is attributed to its inhibitory effect on TNF-α in (22). Recently, the neuroprotective effect of curcumin in traumatic spinal cord injury in mice has been demonstrated to be exerted through the suppression of TNF-α, IL-1β, and IL-6 (23). Furthermore, curcumin has exerted a noticeable suppressive effect on microglial cells in animal models of neurodegenerative diseases (24). These studies provide evidence for the effectiveness of curcumin in attenuation of neuropathological processes in which glial cell and inflammatory cytokines play a crucial role.

Considering the role of glial cells and inflammatory cytokines in the development of morphine dependence along with the inhibitory effects of curcumin on glia cells and inflammatory cytokines, we hypothesized that the implementation of curcumin might attenuate physical dependence to morphine. 

Accordingly, the main goal of this research was to explore the attenuating effects of curcumin on morphine dependence and also investigating the involvement of microglial cells and inflammatory cytokines in the spinal cord. 

## Experimental

*Chemicals*

Curcumin (Sigma–Aldrich Inc., St Louis MO, USA), morphine (Temad Co., Iran), naloxone (Merck Co.), and dimethylsulfoxide (DMSO, Merck Co) were used in this study. The solutions were prepared by solving the chemicals in the normal saline freshly. Dimethylsulfoxide (DMSO) was used as the co-solvent to suspend curcumin in normal saline. A combination of normal saline and DMSO were used as the vehicle.

*Animals*

Male Wistar rats (weighing 250-300 g) were kept under optimum environmental conditions including the temperature of 22 ± 2 °C and a 12 h light-dark cycle. They had access to tap water and food freely. All the experiments of this study were carried out in accordance with the EU Directive 2010/63/EU for laboratory animals. The procedures which were implemented in this study were approved by the ethical committee of Shahid Beheshti University of Medical Sciences (# IR.SBMU.MSP.REC.1395.379).

*Induction of physical dependence*

The animals were divided into five groups (n = 5 in each group; total numbers = 25) including control group, vehicle group, and three different curcumin-treated groups. In order to induce morphine dependence, all the animals in control and curcumin groups were given morphine sulfate intraperitoneally (i.p.) once daily for 9 days in an increasing dose of 10, 20, and 40 mg/kg, with each dose given for 3 days (25). The administration of morphine with a dosage of 40 mg/kg, i.p. was continued for a further 9 days. During the days 10^th^ to 18^th^ control group was given normal saline together with DMSO while curcumin-treated groups received three different doses of curcumin including 2.5, 5, and 10 mg/kg. In the vehicle group, the rats were served as intact animals and neither received morphine nor curcumin. Instead, the animals of the vehicle group were given normal saline (as the solvent of morphine) during the days 1^st^ to 9^th^ and received normal saline + DMSO 0.05% (as the vehicle of curcumin) between the days 10^th^ and 18^th^. On the 19^th^ day, six rats were selected randomly from each group for behavioral evaluation of withdrawal syndrome. The rest of the animals were considered for molecular experiments.

*Evaluation of morphine withdrawal synd-rome*

In order to evaluate physical dependence to morphine, the behavioral symptoms of withdrawal syndrome were evaluated in rats. A day after the end of the treatment period (day 19^th^), six animals in each group were given naloxone (3 mg/kg, i.p.) as the opioid receptor antagonist. The rats were immediately placed in a transparent Plexiglas cylinder (30 cm in diameter and 50 cm height) and the behavioral symptoms of withdrawal syndrome including jumping, leaning, and diarrhea were evaluated in animals for 30 min (16).

*Measurement of inflammatory cytokines by ELISA test*

In order to determine the spinal levels of inflammatory cytokines, the enzyme-linked immunosorbent assay (ELISA) method was utilized. At the end of treatment period (day 19^th^), the other 4 animals in each group were served for measurement of inflammatory cytokines including IL-6 and TNF-α. First, the rats were sacrificed under anesthesia (ketamine 50 mg/kg, i.p., xylazine 5 mg/kg, i.p.) and the lumbar parts of the spinal cord of animals were removed immediately. Then, the concentration of protein in the spinal samples was determined by using the Bradford protein assay based on the method described previously. After the determination of protein contents in spinal samples, the concentration of IL-6 and TNF-α in the lumbar part of the spinal cord was measured by using a sandwich ELISA method. The procedure was conducted in accordance with the manufacturer’s instructions (ab100785 kit, Abcam and RAB 0312-1KT, sigma). An AQ8 ELISA reader (Biotech, USA) was used to determine the amount of absorbance in the standard solutions and spinal samples. The concentration (pg/g protein) of IL-6 and TNF-α in the sample was measured by using standard curves (26).

*Spinal tissue preparation for Iba1 immuno-staining*

 Four remaining rats from each group were sacrificed under anesthesia at the end of the trial (19^th^ day). Then, the lumbar part of the spinal cord was dissected out from rats and transferred to formalin solution 4% in Phosphate-buffered saline (PBS). The spinal tissues were kept in the refrigerator (4 °C) for one week. Then, the samples were transferred to tissue processors for dewatering. After 16 hours, the samples were placed in tissue cassettes and were embedded in paraffin. Finally, the paraffin blocks of samples were cut coronally into 5 μm slices and were placed on the microscope’s slides. In order to determine the activation of microglial cells, the ionized calcium-binding adapter molecule 1 (Iba1) was stained by using the Anti-Iba1 antibody according to the manufacturer’s instructions (Abcam). Briefly, the samples were washed (2 × 5 min) in TBS (tris-buffered saline) plus 0.025% Triton X-100 with gentle agitation. Then, the slides were incubated in 10% normal saline with 1% BSA (bovine serum albumin) in TBS for 2 hours at room temperature. After that, the slides were drained for a few seconds and then wiped around with tissue paper. In the following, the samples were incubated with primary antibody overnight at 4°C. The slides were rinsed (2 × 5 min) with TBS plus 0.025% Triton X-100 and then incubated in 0.3% H_2_O_2_ in TBS for 15 min. Afterward, the samples were incubated with a secondary antibody for an hour at room temperature. At the end of staining, 4′,6-diamidino-2-phenylindole (DAPI) was used to identify the neurons’ nucleus. The slides were placed in DAPI for 20 min at room temperature and eventually washed with water for 5 minutes (27). Finally, immunofluorescence microscopy was implemented to evaluate the intensity of immunostaining of microglial cells in the spinal samples. DAPI (4′,6-diamidino-2-phenylindole) staining was used to identify neurons’ nuclei. 

*Data analysis*

The data were expressed as mean ± SEM for each group. Statistical analysis was carried out using one-way ANOVA, followed by Tukey’s test to compare the mean of each group with the mean of every other group. P < 0.05 was considered significant for statistical analysis.

## Results

*Effects of curcumin on withdrawal syndrome in morphine-dependent rats*

**Figure 1:**
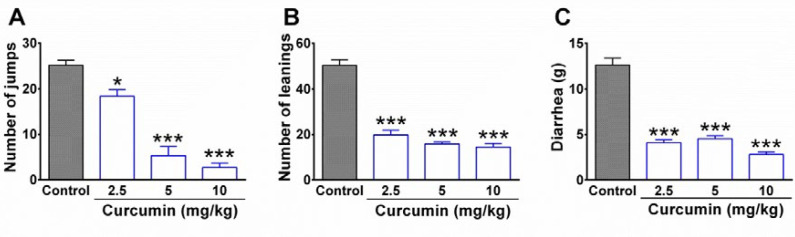
The effect of curcumin on withdrawal syndrome in morphine-dependent rats. (A) Jumping, (B) leaning, and (C) diarrhea were evaluated as behavioral symptoms of withdrawal syndrome over 30 min. Naloxone (3 mg/kg, i.p.) was used to precipitate withdrawal syndrome in rats at the end of the trial (19^th^ day). Data are expressed as mean ± S.E.M for six rats. *P < 0.05, ***P < 0.001 (compared to control). Control: normal saline + DMSO

The results obtained from the behavioral tests showed that the injection of naloxone (3 mg/kg, i.p.) elicited a remarkable withdrawal syndrome in morphine-dependent rats in control groups. As it is shown in Figure 1, a high rate of jumping and leaning was observed in the control group. In contrast, curcumin with dosage of 2.5, 5, and 10 mg/kg significantly decreased the number of jumping (P < 0.05, P< 0.001, and P < 0.001, respectively) and leaning (P < 0.001). In addition, curcumin significantly decreased diarrhea in morphine-dependent rats compared to the control group (P < 0.001; Figure 1C). The injection of naloxone in the vehicle group did not precipitate any jump as the main symptom of withdrawal syndrome (data not shown).

*Effect of curcumin on spinal levels of inflammatory cytokines*

**Figure 2:**
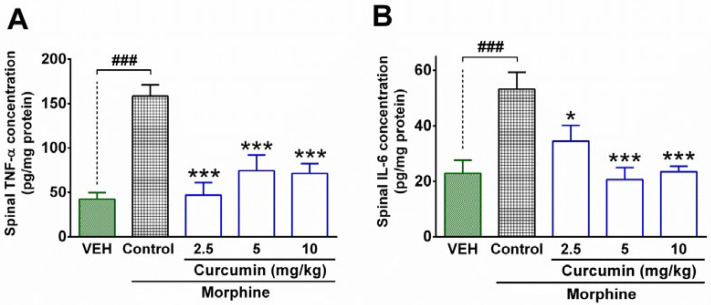
The effect of curcumin on spinal levels of inflammatory cytokines in morphine-dependent rats. The concentration of TNF-α and IL-6 were measured in the lumbar part of the spinal cord by using the ELISA method at the end of the trial (19^th^ day). Data are expressed as mean ± S.E.M for four rats. *P < 0.05, ***P < 0.001 (compared to control); ###*P* < 0.001; Control: normal saline + DMSO

The results attained from the ELISA test showed that the spinal concentration of IL-6 and TNF-α in the control group were significantly higher than the vehicle group (P < 0.001; Figures 2A and B). In contrast, curcumin (2.5, 5, and 10 mg/kg) significantly diminished the concentration of TNF-α in the spinal cord of morphine-dependent rats compared to the control group (P < 0.001; Figure 2A). In addition, treatment with curcumin (2.5, 5, and 10 mg/kg) significantly decreased the spinal levels of IL-6 in the morphine-dependent rats compared to control group (P < 0.01, P < 0.001, and P < 0.001, respectively; Figure 2B). Although, the higher doses of curcumin (5 and 10 mg/kg) resulted in a further decrease in spinal levels of IL-6 (Figure 2B), the difference between groups was not statistically significant. On the other hand, the minimum effective dose of curcumin (2.5 mg/kg) caused a more reduction in spinal levels of TNF-α than the other two doses (Figure 2A); however, the statistical analysis did not indicate a significant difference between aforementioned groups. 

*Effect of curcumin on microglial activation in the lumbar part of the spinal cord*

**Figure 3:**
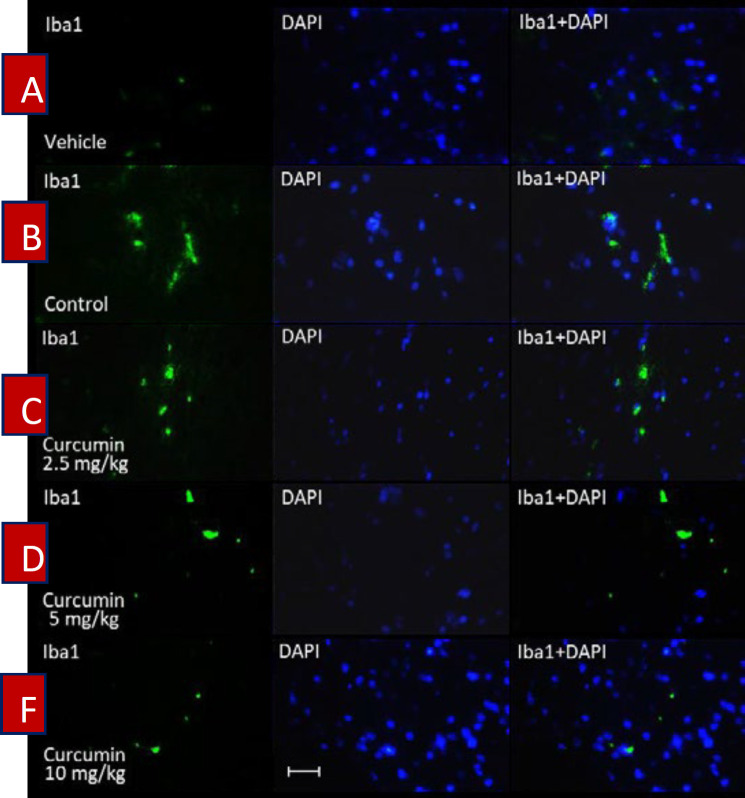
The effect of curcumin on the activation of spinal microglial cells in morphine-dependent rats. Staining was carried out by using Iba1 (green colored) in the lumbar part of the spinal cord. DAPI staining (blue colored) was used to identify neurons’ nuclei. Magnification ×400; Scale bar, 20 micrometer; Iba1, ionized calcium-binding adapter molecule 1; DAPI (4′,6-diamidino-2-phenylindole); Control, normal saline + DMSO

**Figure 4:**
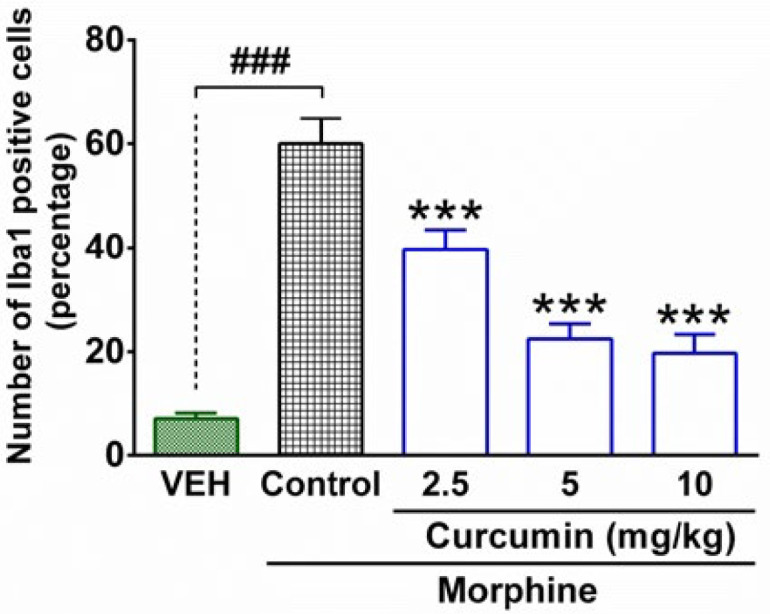
Quantitative analysis of microglial activation in the lumbar part of the spinal cord in morphine-dependent rats. The columns represent the number (%) of Iba1-positive cells in each group. The data are expressed as mean ± SEM for four rats. ***P < 0.001 (compared to control group); ^###^*P* < 0.001; Iba1, ionized calcium-binding adapter molecule 1; Control, normal saline + DMSO, VEH, vehicle

The immunofluorescence microscopy evaluation of spinal samples showed an increase in the intensity of immunostaining by using the Iba1 antibody in the lumbar part of the spinal cord in morphine-dependent rats (Figure 3B). In contrast, treatment of morphine-dependent rats with curcumin (2.5, 5 and 10 mg/kg), diminished the intensity of immunostaining (Figure 3C, D, and F). The statistical analysis of immunofluorescent micrographs by using (Image J) revealed a significant enhancement in the number of Iba1 positive cells in the spinal cord of rats which were subjected to morphine dependence (P < 0.001, compared to vehicle group; Figure 4). On the other hand, curcumin with the dosage of 2.5, 5, and 10 mg/kg caused a significant decrease in the number of Iba1 positive cells in the lumbar part of spinal cord of morphine-dependent rats (P < 0.001, compared to control; Figure 4).

## Discussion

In the current study, the attenuating effect of curcumin on physical dependence induced by long-term administration of morphine was investigated in rats. Our results clearly showed that the attenuating effect of curcumin is mediated via the suppression of activated microglial cells and a remarkable decline in the concentration of inflammatory cytokines in the spinal cord. These findings are consistent with the results of previous studies, such as Ghaemi et al. (28) indicating the attenuating effect of curcumin on withdrawal syndrome. The effects of curcumin on morphine dependence were found to be dose-dependent; because the more increase in curcumin dosage, the further diminution was observed in the dependence symptoms and signs. Although, curcumin was administered intraperitoneally, the findings obtained from behavioral and molecular assays indicated that curcumin cross the blood-spinal barrier proper enough to attenuate morphine dependence. There is evidence indicating that curcumin does not exhibit an optimum pharmacokinetic profile particularly with respect to absorption (29); however, the once-daily administration schedule for curcumin which was executed in the present study led to a reasonable therapeutic effect on morphine dependence. At the same time, curcumin did not cause any adverse effects or mortality in rats over a 9-day period administration. This indicates that curcumin possesses a low toxicity and is well tolerated; however, further investigations should be carried out to clarify various facets of curcumin safety. 

Opioid dependence is an adaptive situation with various physiopathological mechanisms and serious consequences, particularly addiction as well as withdrawal syndrome. The main challenge in the treatment of opioid dependence is discovering a pharmacotherapy approach to deal with mechanisms that underlie the acquisition and maintenance of dependency. Considerable studies have focused on the involvement of supraspinal mechanisms in the process of physical dependence and withdrawal syndrome (30). There are critical brain structures such as lateral paragigantocellularis and locus coeruleus located within the rostral ventrolateral medulla and brain stem regions mediating the development of morphine dependence (31, 32). However, less attention has been paid to the spinal mechanisms which might play a role in the physical dependence phenomenon. 

Recent studies have shown that following chronic administration of opioid agents, a remarkable increase in the activity of microglial cells occurs in the spinal cord (9, 11, 12). This evidence is supported by the fact that the spinal microglial cells do express μ-opioid receptors and their stimulation by various μ-opioid receptor agonists elicits the process of physical dependence (6, 9, 10). In addition, it has been shown that the expression of glial cell-derived neurotrophic factor (GDNF) genes has been increased in the spinal cord of morphine-dependent rats (33). These findings provide further evidence indicating the involvement of spinal microglial cells in the process of opioid dependence. In these conditions, a transition stage from a surveillance status to an enhanced-activity mode occurs in microglial cells which make them capable of secretion a prominent amount of inflammatory cytokines (12). Meanwhile, overexpression of Iba1 has been discovered as a key feature in the process of microglial activation (34). 

Consistent with these studies, our results showed that the induction of morphine dependence in rats was concomitant with a significant rise in the number of Iba1 positive microglial cells in the spinal cord. In addition, our findings from the ELISA test strongly reinforced the results of previous studies indicating the overproduction of inflammatory cytokines following exposure to morphine; while in animals that only received the vehicle, a very low concentration of TNF-α and IL-1β was detected in the spinal samples. In contrast, the treatment of rats with curcumin remarkably suppressed the activated microglial cells which were evident in immunofluorescence staining of spinal samples. This finding is consistent with a number of recent studies showing that curcumin has a potent suppressive effect on microglial cells in different animal models of neurodegenerative diseases (23). It should be noted that long-term consumption of opioid agonists may also cause an increase in the activation of astrocytes (35). On the other hand, there is evidence indicating that curcumin is capable to function as a protective agent against oxidative stress in astrocytes (36). However, further investigation is needed to explore the involvement of spinal astrocytes in the attenuating effect of curcumin on morphine dependence.

In the present study, the inhibitory effect of curcumin on microglial cells was completely conformed with the results of ELISA tests. This is consistent with previous studies indicating that curcumin possesses a remarkable suppressive effect on inflammatory cytokines. For instance, it has been shown that curcumin attenuates lipopolysaccharide-induced infl-ammation in epithelial cells through the inhibition of TNF-α, IL-6, and NF-κB (37). Recently, it has been demonstrated that the neuroprotective effects of curcumin in animal models of neuropathic pain and spinal cord injury are mainly exerted through the suppression of TNF-α, IL-1β, and IL-6 in the spinal cord (22, 23). These findings suggest that curcumin has a strong protective effect against neuropathological processes in which inflammatory cytokines have a main role.

The contribution of spinal inflammatory cytokines in the process of acquisition and development of opioid-induced physical dependence has been reported in previous studies (11, 12). In this regard, it has been demonstrated that morphine-induced over-expression of IL-1β in the spinal cord is strongly related to the somatic symptoms of dependency (14). Moreover, there is evidence indicating a similar relationship between IL-6 and opioid dependence (12). In keeping with these reports, our results obtained from immunostaining and ELISA tests showed good consistency with the findings of behavioral assays in which curcumin attenuated the symptoms of morphine withdrawal syndrome effectively. These observations indicate that the attenuating effect of curcumin on morphine dependence is mediated through the inhibition of spinal inflammatory cytokines. Our findings along with the results from the other studies emphasize the notion that the process of overexpression of inflammatory cytokines in the spinal cord can be considered as a critical stage in the pathology of opioid dependence. 

Nevertheless, other possible mechanisms with respect to the attenuating effect of curcumin on morphine dependence should not be ruled out. For instance, Toll-like receptor 4 (TLR4)-related signaling pathway including NF-κB and p38MAPK/PKC have been recognized as the underlying mechanisms in the course of microglial activation following morphine administration (38). Recent studies have shown that curcumin has a suppressing effect on NF-κB and p38MAPK/PKC pathway (39, 40) and acts as a TLR4 receptor inhibitor (41). Thus, there is a possibility that the signaling pathways through TLR-4 receptors are involved in the attenuating effect of curcumin on morphine dependence; however, this probable mechanism deserves further investigations.

## Conclusion

In conclusion, the results of this study demonstrated that curcumin exerts an attenuating effect on physical dependence induced by long-term administration of morphine in rats. The current study provides evidence indicating that the therapeutic effect of curcumin on morphine dependence is mediated through the suppression of activated microglial cells and the reduction of inflammatory cytokines levels in the spinal cord. Future works are needed regarding other mechanisms of curcumin effect in morphine dependence.

## References

[1] Pasternak GW, Pan YX (2013). Mu opioids and their receptors: evolution of a concept. Pharmacol. Rev..

[2] Martell BA, O’connor PG, Kerns RD, Becker WC, Morales KH, Kosten TR, Fiellin DA (2007). Systematic review: opioid treatment for chronic back pain: prevalence, efficacy, and association with addiction. Ann. Intern. Med.

[3] Kosten TR, O’connor PG (2003). Management of drug and alcohol withdrawal. New Engl. J. Med.

[4] Blevins CE, Abrantes AM, Kurth ME, Gordon AL, Stein MD (2018). Quality of life and well-being following inpatient and partial hospitalization treatment for opioid use disorder. Arch. Psychiatr. Nurs.

[5] Matthes HW, Maldonado R, Simonin F, Valverde O, Slowe S, Kitchen I, Befort K, Dierich A, Le Meur M, Dollé P, Tzavara E (1996). Loss of morphine-induced analgesia, reward effect and withdrawal symptoms in mice lacking the µ-opioid-receptor gene. Nature.

[6] Liu WT, Han Y, Liu YP, Song AA, Barnes B, Song XJ (2010). Spinal matrix metalloproteinase-9 contributes to physical dependence on morphine in mice. J. Neurosci.

[7] Mansour A, Fox CA, Akil H, Watson SJ (1995). Opioid-receptor mRNA expression in the rat CNS: anatomical and functional implications. Trends Neurosci.

[8] Corder G, Tawfik VL, Wang D, Sypek EI, Low SA, Dickinson JR, Sotoudeh C, Clark JD, Barres BA, Bohlen CJ, Scherrer G (2017). Loss of μ opioid receptor signaling in nociceptors, but not microglia, abrogates morphine tolerance without disrupting analgesia. Nat. Med.

[9] Hutchinson MR, Bland ST, Johnson KW, Rice KC, Maier SF, Watkins LR (2007). Opioid-induced glial activation: mechanisms of activation and implications for opioid analgesia, dependence, and reward. Sci. World J.

[10] Mattioli TA, Leduc-Pessah H, Skelhorne-Gross G, Nicol CJ, Milne B, Trang T, Cahill CM (2014). Toll-like receptor 4 mutant and null mice retain morphine-induced tolerance, hyperalgesia, and physical dependence. PloS One.

[11] Raghavendra V, Rutkowski MD, DeLeo JA (2002). The role of spinal neuroimmune activation in morphine tolerance/hyperalgesia in neuropathic and sham-operated rats. J. Neurosci.

[12] Raghavendra V, Tanga FY, DeLeo JA (2004). Attenuation of morphine tolerance, withdrawal-induced hyperalgesia, and associated spinal inflammatory immune responses by propentofylline in rats. Neuropsychopharmacology.

[13] Chao CC, Gekker G, Sheng WS, Hu S, Tsang M, Peterson PK (1994). Priming effect of morphine on the production of tumor necrosis factor-alpha by microglia: implications in respiratory burst activity and human immunodeficiency virus-1 expression. J. Pharmacol. Exp. Ther.

[14] Liu L, Coller JK, Watkins LR, Somogyi AA, Hutchinson MR (2011). Naloxone-precipitated morphine withdrawal behavior and brain IL-1β expression: comparison of different mouse strains. Brain Behav. Immun.

[15] Hosseinzadeh H, Parvardeh S, Masoudi A, Moghimi M, Mahboobifard F (2016). Attenuation of morphine tolerance and dependence by thymoquinone in mice. Avicenna J. Phytomed.

[16] Parvardeh S, Moghimi M, Eslami P, Masoudi A (2016). α-Terpineol attenuates morphine-induced physical dependence and tolerance in mice: role of nitric oxide Iran. J. Basic Med. Sci.

[17] Kunnumakkara AB, Bordoloi D, Padmavathi G, Monisha J, Roy NK, Prasad S, Aggarwal BB (2017). Curcumin, the golden nutraceutical: multitargeting for multiple chronic diseases. Br. J. Pharmacol.

[18] Motaghinejad M, Bangash MY, Hosseini P, Karimian SM, Motaghinejad O (2015). Attenuation of morphine withdrawal syndrome by various dosages of curcumin in comparison with clonidine in mouse: possible mechanism. Iran. J. Med. Sci.

[19] Motaghinejad M, Karimian M, Motaghinejad O, Shabab B, Yazdani I, Fatima S (2015). Protective effects of various dosage of Curcumin against morphine induced apoptosis and oxidative stress in rat isolated hippocampus. Pharmacol. Rep.

[20] Hu X, Huang F, Szymusiak M, Liu Y, Wang ZJ (2015). Curcumin attenuates opioid tolerance and dependence by inhibiting Ca2+/calmodulin-dependent protein kinase II α activity. J. Pharmacol. Exp. Ther.

[21] Cho JW, Lee KS, Kim CW (2007). Curcumin attenuates the expression of IL-1β, IL-6, and TNF-α as well as cyclin E in TNF-α-treated HaCaT cells; NF-κB and MAPKs as potential upstream targets. Int. J. Mol. Med.

[22] Li Y, Zhang Y, Liu DB, Liu HY, Hou WG, Dong YS (2013). Curcumin attenuates diabetic neuropathic pain by downregulating TNF-α in a rat model. Int. J. Med. Sci.

[23] Zhang N, Wei G, Ye J, Yang L, Hong Y, Liu G, Zhong H, Cai X (2017). Effect of curcumin on acute spinal cord injury in mice via inhibition of inflammation and TAK1 pathway. Pharmacol. Rep.

[24] Liu Z, Ran Y, Huang S, Wen S, Zhang W, Liu X, Ji Z, Geng X, Ji X, Du H, Leak RK (2017). Curcumin protects against ischemic stroke by titrating microglia/macrophage polarization. Front. Aging. Front. Neurosci.

[25] Marghmaleki VS, Alaei HA, Malekabadi HA, Pilehvarian A (2013). Effect of physical activity on symptoms of morphine addiction in rats, after and before of lesion of the mpfc area. Iran. J. Basic Med. Sci.

[26] Naderi Y, Sabetkasaei M, Parvardeh S, Zanjani TM (2017). Neuroprotective effect of minocycline on cognitive impairments induced by transient cerebral ischemia/reperfusion through its anti-inflammatory and anti-oxidant properties in male rat. Brain Res. Bull.

[27] Wang J, Li L, Wang Z, Cui Y, Tan X, Yuan T, Liu Q, Liu Z, Liu X (2018). Supplementation of lycopene attenuates lipopolysaccharide-induced amyloidogenesis and cognitive impairments via mediating neuroinflammation and oxidative stress. J. Nutr. Biochem.

[28] Ghaemi-Jandabi M, Abdollahi H, Azizi H, Sadeghizadeh M, Semnanian S (2015). Dendrosomal curcumin nanoformulation attenuates naloxone precipitated morphine withdrawal signs in rats. J. Addiction Res. Ther.

[29] Anand P, Kunnumakkara AB, Newman RA, Aggarwal BB (2007). Bioavailability of curcumin: problems and promises. Mol. Pharm.

[30] Bailey CP, Connor M (2005). Opioids: cellular mechanisms of tolerance and physical dependence. Curr. Opin. Pharmacol.

[31] Ghaemi-Jandabi M, Azizi H, Ahmadi-Soleimani SM, Semnanian S (2017). Intracoerulear microinjection of orexin-A induces morphine withdrawal-like signs in rats. Brain Res. Bull.

[32] Ahmadi-Soleimani SM, Azizi H, Gompf HS, Semnanian S (2017). Role of orexin type-1 receptors in paragiganto-coerulear modulation of opioid withdrawal and tolerance: a site specific focus. Neuropharmacology.

[33] Zhou W, Liu H, Xie X (2000). The expression of glial cell derived neurotrophic factor and its receptor GDNFR-alpha and GDNFR-beta mRNA in spinal cord, brainstem and frontal cortex during morphine withdrawal in rats. Zhonghua yi xue za zhi.

[34] Nakajima K, Kohsaka S (2001). Microglia: activation and their significance in the central nervous system. J. Biochem.

[35] Narita M, Miyatake M, Suzuki M, Kuzumaki N, Suzuki T (2006). Role of astrocytes in rewarding effects of drugs of abuse. Nihon shinkei seishin yakurigaku zasshi= Jpn. J. Psychopharmacol.

[36] Daverey A, Agrawal SK (2018). Pre and post treatment with curcumin and resveratrol protects astrocytes after oxidative stress. Brain Res.

[37] Sun H, Chen X, Han Z (2017). Effects of curcumin on the inflammatory reaction induced by LPS and the expression of SIGIRR in alveolar epithelial cells. J. Clin. Med.

[38] Su M, Ran Y, He Z, Zhang M, Hu G, Tang W, Zhao D, Yu S (2018). Inhibition of toll-like receptor 4 alleviates hyperalgesia induced by acute dural inflammation in experimental migraine. Mol. Pain.

[39] Yang Z, Zhao T, Zou Y, Zhang JH, Feng H (2014). Curcumin inhibits microglia inflammation and confers neuroprotection in intracerebral hemorrhage. Immunol. Lett.

[40] Yu Y, Shen Q, Lai Y, Park SY, Ou X, Lin D, Jin M, Zhang W (2018). Anti-inflammatory effects of curcumin in microglial cells. Front. Pharmacol.

[41] Chen CY, Kao CL, Liu CM (2018). The cancer prevention, anti-inflammatory and anti-oxidation of bioactive phytochemicals targeting the TLR4 signaling pathway. Int. J. Mol. Sci.

